# Reflections on Pandemic (H1N1) 2009 and the International Response

**DOI:** 10.1371/journal.pmed.1000346

**Published:** 2010-10-05

**Authors:** Gabriel M. Leung, Angus Nicoll

**Affiliations:** 1 Food and Health Bureau, Government of the Hong Kong SAR, People's Republic of China; 2 European Centre for Disease Prevention and Control, Stockholm, Sweden

## Abstract

Gabriel Leung and Angus Nicoll provide their reflections on the international response to the 2009 H1N1 influenza pandemic, including what went well and what changes need to be made in anticipation of future flu pandemics.

**Summary Points**

- Many of the initial responses to the 2009 H1N1 pandemic went well but there are many lessons to learn for future pandemic planning.
- Clear communication of public health messages is crucial, and should not confuse what could happen (and should be prepared for) with what is most likely to happen.
- Decisions regarding pandemic response during the exigencies of a public health emergency must be judged according to the best evidence available at the time.
- Revising pandemic plans—to be more flexible and more detailed—should wait for WHO leadership if national plans are not to diverge. Surveillance beyond influenza should be stepped up, and contingencies drawn up for the emergence or re-emergence of other novel and known pathogens.
- Data collection and sharing are paramount, and include epidemiological and immunological data. Clinical management of severe influenza disease should not be limited to the current antiviral regimen, and include the development of other therapeutics (e.g., novel antivirals and immunotherapy).
- Greater and more timely access to antivirals and influenza vaccines worldwide remains an ongoing challenge.

## What Happened?

There is general consensus that the only predictable characteristic of influenza viruses and pandemics is their unpredictability [1]. Given such uncertainty, reasonable application of the precautionary principle should prevail in the responses. Indeed many of the initial responses to the 2009 pandemic went well. Once isolated, the pandemic virus strain was shared immediately, specific diagnostic assays were produced and distributed worldwide, antivirals were available in many countries, vaccine development started promptly, and clinical trials demonstrating vaccine safety and immunogenicity were conducted rapidly.

There were many inherently favourable features of the pandemic itself, not all of which were immediately apparent (Table 1). This was not 1918 Spanish flu. The impact has been mostly confined to the health sector. But that impact has been significant and heterogeneous, with pressure experienced by primary and hospital care (especially intensive care and paediatric services). Distilling descriptions of the impact of a complex public health threat like a pandemic into a single term like “mild,” “moderate,” or “severe” can potentially be misleading [2]. Certainly the experience of hospital clinicians indicated that this pandemic, sometimes described as “mild to moderate,” was not limited to only mild or moderate illness. Many patients were severely ill and died, and undoubtedly, high-quality clinical management of patients with severe complications in intensive care units saved many lives of the critically ill, who often required prolonged hospitalisation [3].

**Table 1:** What has occurred in this pandemic and what could have been worse.

What Has Occurred to Date	What Could Have Been Worse
A pandemic virus strain was first detected in North America	Emergence in a less developed setting
Immediate virus sharing following virus isolation to promote rapid development and deployment of available diagnostics and vaccine candidates	Delayed virus sharing
Apparent lower global impact in terms of disease severity on an overall population basis (i.e., all ages) compared to other pandemics (e.g., 1918 H1N1 virus), and to what was feared (e.g., HPAI H5N1 virus)	A more pathogenic pandemic virus, or rapid evolution of more virulent circulating strains
Residual immunity among a proportion of the population (i.e., older adults)	Total population susceptibility
Circulating pandemic virus strains susceptible to neuraminidase inhibitor antivirals stockpiled for pandemic planning and available in some countries	Widespread neuraminidase inhibitor resistance in circulating strains; no antiviral medications available
Rapid dissemination of epidemiological, clinical, and virological data from North America and the Southern Hemisphere to inform the global community	Poor or incomplete data, or lack of transparency and information sharing
Emergence during the end of a seasonal influenza epidemic	Emergence during the peak of a seasonal influenza epidemic to complicate disease and virus identification and increase pressures on health services
Mild uncomplicated illness in most people with pandemic influenza virus infection	A more pathogenic virus causing a high frequency of severe complications
Basic reproductive number (*R*_o_) on a similar scale to seasonal influenza virus transmission	A higher *R*_o_
Modes of transmission similar to seasonal influenza A and B virus spread	Different modes of transmission (e.g., substantial contact/fomites transmission, conjunctival/ocular infection)
A vaccine that is highly immunogenic usually requiring only a single injection	Poor immunogenicity and requiring multiple injections
A vaccine that appears to be safe and with similar safety profile to seasonal influenza vaccine, with very low frequency of severe complications	Frequent and severe vaccine-associated adverse events

The epidemiology of this pandemic is different than for seasonal influenza epidemics, but not unlike previous pandemics. Young people have been disproportionately affected in terms of hospitalisation and deaths compared to seasonal influenza in which complications and mortality are predominantly borne by the elderly [4]. Similarly, the risk to pregnant women has been higher than for seasonal influenza [5],[6], which was also noted in previous pandemics. The attributable premature mortality may remain unclear for some time. Recent American analyses have estimated many more deaths than those officially reported with laboratory confirmation of infection and that years of life lost were equivalent to the 1968 pandemic. The lower bound of such estimates is equivalent to the annual burden caused by a typical H3N2 seasonal epidemic in temperate climates [7],[8]. The years-of-life-lost metric captures the impact of a different age-specific mortality pattern which death counts cannot. Deaths involving the young and healthy incur many more potential years of life lost compared to those of older adults and of chronically ill individuals.

There are also a number of “firsts” for the 2009 pandemic after an interpandemic period of more than four decades (Box 1). These brought both opportunities and challenges. Under the auspices of the World Health Organization (WHO), the process of a global review by public health specialists from around the world has recently begun. They were nominated by national authorities and are led by an elected chair who assessed the handling of the 1976 swine influenza event among US military personnel at Fort Dix [9]. Here we offer some initial reflections on the first 12 months of the present pandemic.

**Box 1. A Series of “Firsts” about Pandemic (H1N1) 2009**

- The first pandemic to emerge in the twenty-first century. It has been more widespread and remains ongoing, compared to SARS.
- The first pandemic to occur after major global investments in pandemic preparedness had been initiated.
- The first pandemic for which effective vaccines and antivirals were widely available in many countries, thus requiring public health authorities to earn and retain the confidence of health care providers through whom such are usually distributed.
- The first influenza pandemic to coincide with the ongoing HIV/AIDS pandemic and for which preliminary data do not suggest a substantial, disproportionate impact on HIV-infected patients.
- The first pandemic that took place within the context of a set of International Health Regulations and global governance, which had not been widely tested until the present.
- The first pandemic with early diagnostic tests that led to rapid diagnosis but also an early obsession in the media and of policymakers with having reports of the numbers of those infected.
- The first pandemic with antivirals available in many countries that led to a hopeful expectation that the pandemic might be containable, leading to the preparation for and implementation of a “containment phase” in some places.
- The first pandemic in which intensive care was available in many countries to treat critically ill patients, fostering an expectation that everyone could be treated and cured.
- The first pandemic with instant communication so that early impressions (such as the experience and response in Mexico and the Ukraine) could be shared ahead of proper scientific analysis.
- The first pandemic in which web-based platforms of traditional journals expedited dissemination, complemented by other innovative online resources (e.g. *PLoS Currents: Influenza*, http://knol.google.com/k/plos-currents-influenza#, based on Google's knol technology).
- The first pandemic with a “blogosphere” and other rapid social media messaging tools that challenged conventional public health communication.

## Surveillance

Considerable effort in recent years had been dedicated to preparing for surveillance during a pandemic and to incorporating modelling in planning in some countries. The pandemic virus was detected and isolated reasonably early, although too late for any attempt at containment. It remains unclear precisely when or where it first emerged, but the earliest human infections were detected in North America and the best estimates of the timing of emergence are variously mid-February from field epidemiology in southeast Mexico or mid-January from a molecular clock model [10]. Situational awareness during the early phase allowed quick assessment by countries, notably those affected first (Mexico, US, Canada, and Southern hemisphere temperate countries). The integration of clinical, laboratory, and epidemiologic data proved essential and gave important insights into disease severity, transmission dynamics, and anticipated impact of interventions. Focused local or national studies with analyses shared through WHO or regional bodies proved more valuable than relying on collection of primary data for analysis in some regions [2]. Although there were modelling efforts underway, only a few governments incorporated such data for policy decisions.

## Seroepidemiology

Data from seroepidemiological studies have been limited, primarily due to the lack of routine influenza serosurveys, and technical challenges with the assays, interpretation, and validation of results. Available serological data on prevalence or seroincidence of humoral immunity yielded age-specific attack rates that indicated a substantial proportion of asymptomatic infections and mild illnesses, similar to or greater than past pandemics and seasonal outbreaks. This was confirmed by a recent Hong Kong study showing the proportion of asymptomatic infection, secondary attack rates, viral shedding, and course of illness among household members were largely similar between infections with seasonal and pandemic influenza virus strains circulating during 2009 [11]. The few published serosurveys revealed heterogeneities in infection rates among different groups and between different places [12]–[14]. In particular there appears to be serological evidence of substantial preexisting humoral immunity among older adults, ranging from 23% (1∶32 titre by haemagglutination inhibition in those 65 years or over) [14] to 34% (1∶80 titre by microneutralisation assay in those 60 years or over) [15] in different studies. Further data on population susceptibility by age or the availability of a rapid and accurate serological test could allow health services to further target vaccine efforts for subsequent waves, as has been done in a few countries [14].

## Nonpharmaceutical Interventions

Early on, some airports installed thermal screening and others asked travellers to declare fever or respiratory symptoms at disembarkation. The utility of these interventions has been repeatedly challenged [16], although if executed well could delay the start of community transmission by a few weeks [15],[17] (Table 2). Similarly, during the early stages of global or local spread, quarantine, isolation, school closures, and other social distancing measures were variously implemented in some populations (e.g., Mexico [18], western Japan [19], UK), although most have not yet been formally evaluated and published [20]. Two exceptions are in Hong Kong and the UK. In the former, it was estimated that transmission fell by 25% when schools closed [21]. In settings like Hong Kong, with the infrastructure and resources to implement such measures and a populace sensitised by the 1997 H5N1 and 2003 SARS experiences, not carrying out border screening and social distancing would have been untenable. It had been felt that containment of a pandemic would be ineffective except perhaps during Phase 4 (WHO definition) [22] but some countries attempted containment in Phases 5 and 6. Some countries even instituted a “containment phase” using case-finding and various measures such as isolation and antiviral treatment of ill suspected and confirmed cases, and quarantine of exposed persons with or without antiviral chemoprophylaxis, while others never attempted or quickly moved from resource-intensive containment to mitigation [22]. A preliminary evaluation of intensive containment undertaken in parts of the UK during its spring/summer wave of 2009 demonstrates how resource- and labour-intensive community containment could have been and also how even with a lot of resources the measures had to be abandoned [23]. It is now recognised that the phrase “containment” was unfortunate and potentially misleading since at best the actions were only mitigating impact [24].

**Table 2:** Objectives and limitations of public health interventions in pandemic (H1N1) 2009.

Intervention	Objective – What It Was Intended For	What It Cannot Do or Was Not Intended to Achieve	Notes	Populations That Implemented the Intervention
**Nonpharmaceutical**				
Border measures: screening, quarantine, and isolation	Reduce and delay community spread somewhat at the earliest stage to allow better preparation for mitigation response [15]	Completely prevent entry of infected individuals due to suboptimal sensitivity and asymptomatic (including infected and within incubation period) or subclinical presentation [16]	Many countries did not attempt these measures because of logistics, stage of pandemic [22] or other cost-benefit considerations [16]	ChinaHong Kong SARJapan
Personal protective measures (e.g., face masks, hand hygiene, cough etiquette, early self-isolation when ill)	Reduce risk of infection to self and close contacts (if self is ill and infected) [27],[28]	Have not been evaluated whether they can provide significant population-level protection	Virtually all countries implemented these measures to varying degrees in health care settings according to the risk of the situation. Almost all encouraged hand hygiene, cough etiquette, and early self-isolation	Most countries recommended adoption of hand hygiene, cough etiquette, and early self-isolation when ill, but use of face masks in the community was uncommon except in East Asia.
**Pharmaceutical**				
Antivirals for treatment and chemoprophylaxis [21],[22]	Mitigation: reduce illness severity and complications if administered early; reduce transmission from those receiving treatment; sometimes also used as chemoprophylaxis in high-risk circumstances	Provide significant population-level protection or allow containment	Attempts at source containment were not possible, as the pandemic was effectively already in WHO Phase 5 when what became the pandemic virus was first identified [22]. Initial observational studies suggest antivirals were successful when early treatment was administered	CanadaGermanyHong Kong SARJapanUKUS(these populations attempted the intervention initially but effort was not sustained towards the later stages of the pandemic)
Vaccines	Mitigation(a) at individual level by conferring immunity to infection in those at higher risk of severe disease or (b) at a population level by immunizing population groups especially those who are transmitting most (i.e., children)		In most countries vaccine was not available early enough and/or arrived in insufficiently large amounts to achieve mitigation at a population level. Greater population benefit may occur in the next season	Most countries of the developed North, especially those with advance purchase agreements

This pandemic virus transmitted efficiently among children and at least one study has shown that school closures were associated with reduced population transmission when implemented early [21]. Closures appear to have stopped school outbreaks in western Japan and might have also mitigated impact initially on the local communities [25]. However, decisions on this intervention were contextually specific, dependent on feasibility and their potential downsides [26]. In Europe and the US the judgement was generally that proactive school closures would not be justified as a community mitigation intervention in the context of a perceived mild-to-moderate pandemic among the general population, and reserve plans for widespread closure have not been activated in most jurisdictions. However, local decisions were made to close schools in some areas as a response to prevent transmission and high attack rates among schoolchildren or simply where there was too much illness and absenteeism to sustain teaching [21],[27].

Personal protective interventions such as face masks, hand hygiene, and early isolation may have been beneficial in reducing transmission at the individual level in the home [27],[28], although household secondary attack rates during the pandemic were similar to those with seasonal influenza [13],[29]. Their population level impact remains to be assessed. There was much debate over whether to use conventional masks or respirators in health care settings. One well-conducted Canadian trial on seasonal influenza virus transmission published during the pandemic suggested no additional advantage from N95 respirators [30].

## Antivirals

Oseltamivir and zanamivir (and later peramivir in some countries) played a role in the mitigation effort, sometimes drawing on national stockpiles. Except for Japan, widespread use of antivirals had not been the norm previously. It became standard to recommend neuraminidase inhibitors for treatment of inpatients and high-risk outpatients, and in restricted circumstances for chemoprophylaxis. Innovative delivery schemes were sometimes developed. Those who fell sick in England could have a telephone assessment (taking pressure off primary care) and then if appropriate receive empiric oseltamivir treatment from a local pharmacist. In Norway oseltamivir was made available “over the counter.” However in many European settings, reluctance remained among primary care providers to prescribe a drug they were unused to. Another controversy was whether to offer oseltamivir to all those with symptoms or target those at higher risk for complications. The observational data so far suggest that early treatment with neuraminidase inhibitors have worked to reduce severe disease and have not been linked to significant adverse risks [31],[32]. Late clinical presentation and delayed initiation of antiviral treatment have been implicated with more severe complications worldwide, indicating gaps in identifying and treating patients before disease severity increases. While sporadic cases of oseltamivir resistance have been reported in association with a specific mutation (H275Y in neuraminidase), such oseltamivir-resistant viruses have rarely transmitted [3]. Indeed, the pandemic virus has remained genetically and antigenically stable so far.

## Vaccines

The core pharmaceutical preventive intervention was vaccines and this has been a particular focus for critics citing the uneven and suboptimal uptake across countries. Development of a pandemic vaccine was a scientific success, but limited availability until after the autumn/winter wave had nearly peaked in the Northern Hemisphere contributed to lower coverage than anticipated [33]. Vaccination coverage depended on many factors, including availability, preordering, licensing and bureaucratic hurdles, logistics, convenience, and, most crucially, public and professional perceptions. This pandemic presented a particular risk communication challenge, since while infection usually results in mild illness, occasionally it is lethal, even in the young and previously healthy despite optimal treatment [34]–[36]. In the absence of any excess risk of serious side effects compared to annual seasonal vaccines [37] (despite the intensive effort to look for such) the benefits of immunisation far outweighed any potential downsides at the individual level, particularly for those at higher risk for complications. Notwithstanding such evidence, the cost of pandemic vaccines was considerable and a loss of public confidence has sometimes been triggered by unsubstantiated media reports of serious side effects with a “new vaccine” that utilised the same manufacturing technology as for years of seasonal vaccines. Uptake among health care providers as role models has been mixed, as has their expression of the need for vaccination at all. This sometimes cast doubt in the minds of the public. Conversely, pandemic deaths in young healthy people abruptly changed public perception (such as in Canada, Romania, and Finland); supply and organisational issues then became crucial.

Another more fundamental criticism challenges whether vaccines should have been procured at all given an eventual surplus in the developed North. The unexpected finding that a single dose was immunogenic among all persons except for younger children, which reduced the required number of doses by half from the projected number needed in most countries, but this was not known in advance of countries placing vaccine orders. Had there been “overpreparation”? The prior worry had been the reverse – would there be sufficient production capacity to meet needs [38]? Even in retrospect, and with the observed burden of the pandemic, a vaccine was clearly justified for countries where annual vaccines for seasonal influenza are routinely recommended.

Field and pharmacovigilance data so far have shown that these vaccines were immunogenic, effective, and very safe [39]. However, the frailty was timing and availability. Generally supplies came in later and in smaller amounts than forecasted, in part due to lower yield in growth of the vaccine virus strain than expected. Most of the orders arrived after the peak of the autumn/winter wave in the geographic north (whose countries had received most vaccines). Therefore, judgement on their impact in averting serious morbidity and deaths may come only after the second winter. Perhaps then, differential use by countries will allow for comparisons where there is good surveillance for severe disease and deaths.

There have been claims of extraneous influence on the independent and objective judgment of expert advice that in turn influenced decision-making [40]. These claims have been robustly countered as they relate to WHO's advisory and decision-making process [41]. As Harvey Fineberg, chair of WHO's external review, pointed out, when assessing any allegations of impropriety or bias, or the perception of such, it would be important to distinguish between financial or other conflicts with potential pecuniary gains versus predispositions arising from an individual's background and experience. Rather than aiming for a complete purge of any and all experts who had worked with vaccine manufacturers and received sponsorship, as these are often the very same group who possess the most relevant and useful expertise precisely because they have been closely involved in the research and development process, the focus should be on making the declaration of such interest wholly transparent and comprehensive according to a set of robustly established procedures that can withstand the strictest scrutiny. It is reassuring that WHO has honoured its commitment to making public the names and declarations of interest of the pandemic Emergency Committee when the pandemic was declared over on 10 August 2010. Additionally, receiving advice should be differentiated from making decisions. The people entrusted with undertaking the latter task should then judge the validity of the advice rendered by experts, having taken into account their interest declarations. The decision makers should also be prepared to justify their actions.

## Looking Forward

It is important to learn from our experience through the first year and beyond as we move into the *new* seasonal influenza [42],[43]. It is theoretically possible, although unlikely, that the second winter of this pandemic will be worse than the first, as happened for the 1968 pandemic when transmissibility increased [44]. Equally, if the pandemic virus out-competed the A(H3N2) virus strains responsible for more intense seasonal epidemics, there may even be a diminution of disease burden in older people. As of this writing, seasonal influenza A (H3N2) and B virus strains continue to cocirculate. Antigenic drift in the 2009 H1N1 virus is expected to occur in the future, especially under the pressure of so many people now being immune through infection or immunisation, although the timing is unpredictable. The pandemic virus is included in the trivalent seasonal influenza vaccine composition for both hemispheres. Clear communication of public health messages will remain a particular challenge and not confusing what could happen (and should be prepared for) with what is most likely to happen. In assessing the pandemic response, decisions made during the exigencies of a public health emergency must be judged according to the best evidence available at the time. Hindsight always gives perfect vision and using post-hoc information to evaluate prior decisions at best confuses and often produces unfair conclusions.

Preparedness plans will have to be revised in due time, after the many lessons learned have been gathered. This should be done quickly in case the worst is not yet over [45]. However, rewriting plans should best wait for WHO leadership if national plans are not to diverge. A strong argument exists for making future plans more flexible and having extra descriptions including the many aspects of severity when a pandemic is emerging that then determine the consequential public health actions [2]. Broadening surveillance for a range of influenza A viruses among a wide range of animals (e.g., swine), not just in avian species, as well as strengthening the monitoring of seasonal influenza virus infections in humans will facilitate identification of novel influenza A viruses of pandemic potential, and earlier detection of the emergence of a pandemic virus. More broadly we should look beyond influenza and draw up contingencies for the emergence or re-emergence of other novel and known pathogens [45].

One challenge faced initially in this pandemic was for timely collection and sharing of clinical data to inform optimal management of critically ill patients worldwide. Establishing clinical research infrastructure prior to a pandemic and a central institutional review board will facilitate data collection and analyses [46], whether for the next influenza pandemic, SARS outbreak, or next novel respiratory pathogen of global importance. Clinical management of severe influenza disease should not be limited to the current antiviral regimen, and include the development of other therapeutics (e.g., novel antivirals and immunotherapy).

Ongoing improvements in the routine and timely monitoring of hospital admissions and deaths attributable to influenza, as well as representative serological surveys at regular intervals can provide epidemiological data with which to reduce uncertainty around the true burden of influenza and thus inform policy choices [47].

Assessment of the humoral and cellular immune response over time in a subset of vaccinated individuals could reveal how vaccine-induced immunity differs from natural infection, and whether cross-reactive responses to other influenza virus strains are modulated by the two types of immunological response [48]. The latter could become important as the pandemic strain has already been cocirculating with other interpandemic influenza A virus strains in some parts of the world.

Greater access to antivirals and influenza vaccines worldwide is an ongoing challenge. Although WHO secured pledges of 200 million vaccine doses and monies for operations, and more than 80 less-resourced countries have signed agreements with WHO for supply of vaccines, this gap remains. It is an indefensible fact that these vaccines started to flow to the poorer countries well after they began going to the countries with advance purchase arrangements. Delivering timely pandemic influenza vaccination in countries without existing seasonal vaccine programmes is proving difficult. The long-term solution has to be improved surveillance, expanded monitoring of disease burden, and better prevention and control of influenza, including the development of seasonal vaccine use and production in all regions of the world [49]. Increased coverage of available bacterial vaccines (Hib, pneumococcal) will help prevent secondary invasive bacterial coinfections with either seasonal or pandemic influenza.

Finally accusations of “overreaction” can be countered by the observation that investment in fire services or insurance is usually judged against their ability to respond to conflagrations. If the first test is a lesser fire, that experience should be used for improvements rather than as a reason to scrap the fire engines and cancel the insurance [40].
